# Codesign and Launch of ‘On the Ball’: An Inclusive Community‐Based ‘Testicular Awareness’ Campaign

**DOI:** 10.1111/hex.14100

**Published:** 2024-06-14

**Authors:** Mohamad M. Saab, Varsha N. Shetty, Megan McCarthy, Martin P. Davoren, Angela Flynn, Ann Kirby, Steve Robertson, Gillian W. Shorter, David Murphy, Michael J. Rovito, Frances Shiely, Josephine Hegarty

**Affiliations:** ^1^ Catherine McAuley School of Nursing and Midwifery University College Cork Cork Ireland; ^2^ Sexual Health Centre Cork Ireland; ^3^ School of Public Health University College Cork Cork Ireland; ^4^ Department of Economics, Cork University Business School University College Cork Cork Ireland; ^5^ School of Allied Health Professions, Nursing & Midwifery, Faculty of Health University of Sheffield Sheffield UK; ^6^ Drug and Alcohol Research Network, School of Psychology Queen's University Belfast Belfast UK; ^7^ School of Computer Science & Information Technology University College Cork Cork Ireland; ^8^ Department of Health Sciences, College of Health Professions and Sciences University of Central Florida Orlando Florida USA; ^9^ HRB Clinical Research Facility University College Cork Cork Ireland

**Keywords:** health promotion, men's health, qualitative research, sexual and gender minorities, testicular diseases, user‐centred design

## Abstract

**Introduction:**

Increased awareness of testicular diseases can lead to early diagnosis. Evidence suggests that men's awareness of testicular diseases is low, with many expressing their willingness to delay help‐seeking for symptoms of concern. The risk of testicular diseases is higher in gender and sexual minority groups. In this study, we discuss the codesign, refinement and launch of ‘On the Ball’, an inclusive community‐based ‘testicular awareness’ campaign.

**Methods:**

The World Café participatory research methodology was used. Individuals from Lesbian, Gay, Bisexual, Transgender and Queer+ friendly organisations, testicular cancer survivors, policymakers, media/marketing experts and graphic designers were recruited. Participants were handed a brief for ‘On the Ball’, which was designed based on feedback from a previous World Café workshop. They were assigned to three tables. Participants rotated tables at random for three 20‐min rounds of conversations. Each table had a facilitator who focussed on one element of the campaign brief. Data were collected using audio recorders and in writing and were analysed thematically.

**Results:**

Thirteen individuals participated in the workshop. The following themes emerged from the data: (i) campaign identity, (ii) campaign delivery and (iii) campaign impact. Participants recommended enhancements to the campaign logo, slogan, social media posts and poster. They suggested delivering the campaign online via social media and offline using various print and broadcast media. Participants recommended targeting areas with a large number of men such as workplaces. To help measure the impact of the campaign, participants proposed capturing social media analytics and tracking statistics relating to testicular diseases. Recommendations were used to refine the ‘On the Ball’ campaign and launch it in a university. In total, 411 students engaged with the various elements of the campaign during the soft launch.

**Conclusions:**

‘On the Ball’ campaign visuals ought to be inclusive. Online and offline campaign delivery is warranted to reach out to a wider cohort. Campaign impact can be captured using social media analytics as well as measuring clinical outcomes relating to testicular diseases. Future research is needed to implement the campaign online and offline, explore its impact and evaluate its feasibility, acceptability, cost and effect on promoting testicular awareness.

**Patient or Public Contribution:**

The ‘On the Ball’ campaign was codesigned and refined with members of Lesbian, Gay, Bisexual, Transgender and Queer+ friendly organisations, testicular cancer survivors, health policymakers, media and marketing experts and graphic designers using the World Café participatory research methodology.

## 1 Introduction

Malignant and benign testicular diseases are most prevalent among men younger than 50 years. The incidence of testicular cancer is on the rise globally [1]. Out of 71,000 new reported testicular cancer cases worldwide in 2018, more than a third were in Europe, with incidence rates ranging from 7.8 to 6.7 per 100,000 men [1]. Testicular cancer is curable with surgical removal of the affected testis, especially when diagnosed early [2, 3].

A painless mass in one of the testes is the most common symptom of testicular cancer. In 80% of cases, this mass is discovered accidentally by men [4, 5]. Benign testicular diseases, such as infections in the epididymis and/or testis, enlargement of the veins in the testis and twisting of the spermatic cord, which provides blood flow to the testis, are more common than testicular cancer and can cause similar symptoms [6, 7, 8, 9, 10]. This highlights the importance of raising men's awareness of testicular diseases and promoting early help‐seeking for self‐discovered signs and symptoms of testicular disease. Indeed, in a study of 215 surgeries where testes affected by testicular cancer were surgically removed between 1975–1985 and 2007–2012, it was found that tumour size was reduced significantly [11]. This was attributed to increased testicular cancer awareness.

Findings from six systematic reviews conducted between 2015 and 2023 indicate that men's awareness of testicular diseases (particularly benign testicular diseases), intentions to seek help for symptoms of concern and behaviours in terms of performing testicular self‐examination were all suboptimal [12, 13, 14, 15, 16, 17]. The latest of these reviews found that several interventions were successful in increasing men's awareness of testicular cancer and testicular self‐examination, including ‘a PowerPoint presentation, an online educational brochure, video‐assisted teaching, a motivational video, and a virtual reality game’ (p. 2) [17]. However, only one study addressed help‐seeking for testicular symptoms and aimed to promote men's awareness of both malignant and benign testicular diseases [18]. It was also noted that none of the included studies focused on gender and sexual minority groups who are at a greater risk of health disparities including testicular diseases [17]. For example, infections of the epididymis and/or testis are often transmitted sexually and are common in the gay community and in men who have sex with men [19, 20].

Increased ‘testicular awareness’ can potentially lead to early presentation and diagnosis of testicular diseases. Testicular awareness involves ‘(i) familiarity with own testes; (ii) knowing what is normal versus abnormal; (iii) ability to detect an abnormality; and (iv) knowing own risk factors’ [21] (p. e3), regardless of the ultimate diagnosis. To help raise testicular awareness while also being inclusive, we conducted a study where we used the World Café (WC) participatory research methodology to codesign an inclusive community‐based testicular awareness campaign. This study is published elsewhere [22].

Participants in our previous study recommended using social media platforms to disseminate the campaign messages. They believed that individuals who do not use social media can be targeted using, for example, radio, television and print media. Participants stressed the importance of addressing the embarrassment that men feel when discussing testicular diseases [22]. They suggested signposting the public to sources of information on these diseases. Participants recommended multimodal, dynamic and phased campaign delivery, beginning with online campaigning to help build the campaign profile and then moving to offline advertising to ensure wider reach. The use of light humour and words like ‘balls’ was perceived as acceptable to help engage younger people [22].

Findings from our previous study were shared with a creative communications agency that helped design the ‘On the Ball’ campaign. This involved preparing a 16‐page brief containing the campaign logo, slogan, overview, poster, messages underpinned by the concept ‘testicular awareness’, sample social media posts and recommended strategies for campaign delivery.

A second WC workshop was held with the same group of participants to discuss the campaign brief and plan for the campaign launch. Findings from the second WC workshop and the soft launch of the ‘On the Ball’ campaign are reported in the present paper. Therefore, the aim of the current study is to discuss the codesign, refinement and launch of ‘On the Ball,’ an inclusive community‐based ‘testicular awareness’ campaign.

## 2 Methods

**Figure 1:**
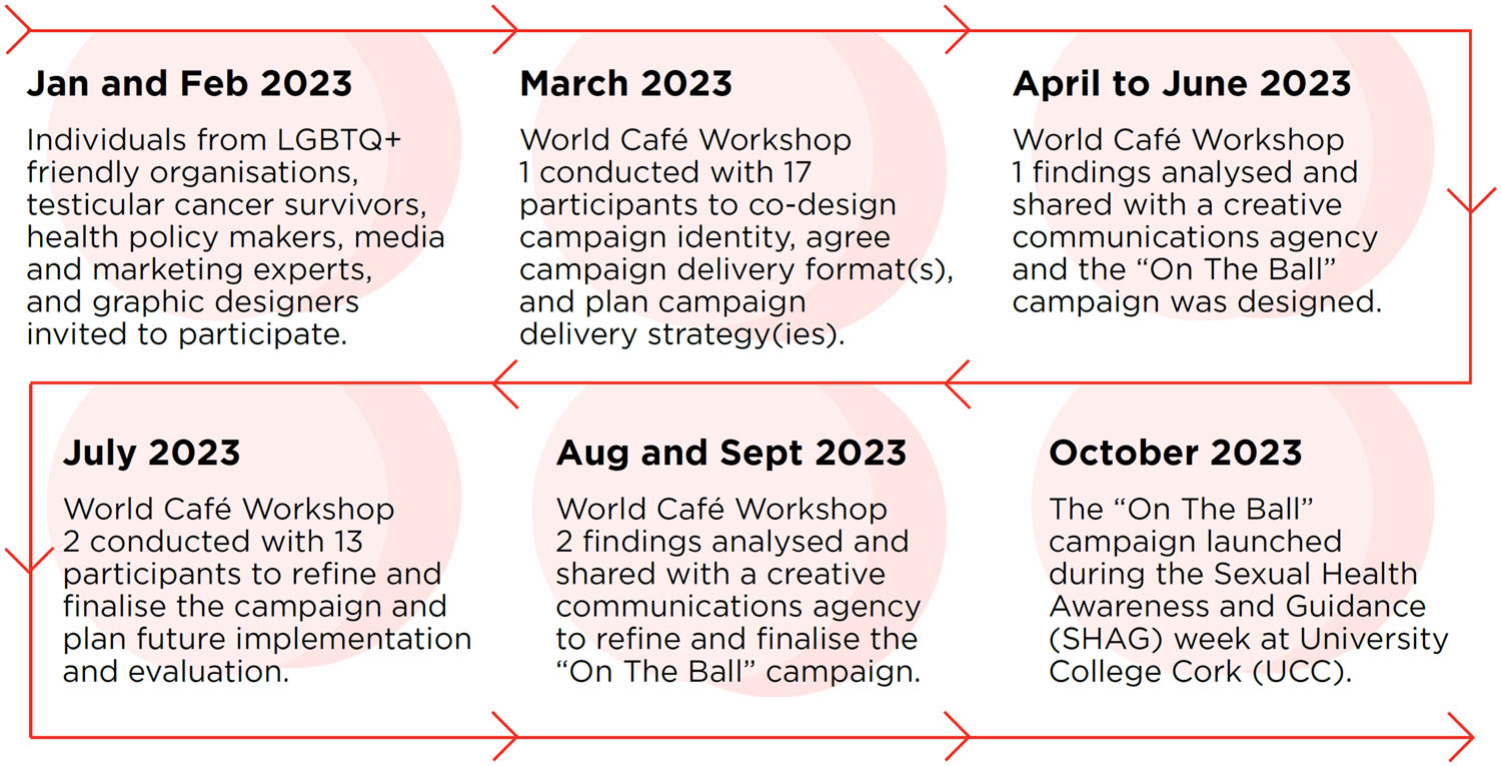
The steps taken to codesign, refine and deliver the ‘On the Ball’ testicular awareness campaign.

The full study process and timelines are summarised in Figure 1. This study was guided by the WC design principles [23, 24, 25]. It received ethical approval from the Social Research Ethics Committee at University College Cork (Log 2022‐227).

### 2.1 Participants and Setting

Purposive sampling was used to recruit adults (aged ≥18 years), residing in the Republic of Ireland, and who were members of Lesbian, Gay, Bisexual, Transgender and Queer+ friendly organisations, testicular cancer survivors, health policymakers, media and marketing experts and/or graphic designers. Individuals were excluded if they did not consent to participate.

Participants in the first WC workshop which took place in March 2023 consented to their e‐mail address being kept on record. They were subsequently contacted via e‐mail and invited to contribute to the second WC workshop. A study information sheet was used to explain the aim of the second WC workshop and assure participants that anonymity would be maintained, there were no risks from their participation, and light refreshments would be served on the day.

### 2.2 Procedures

**Figure 2:**
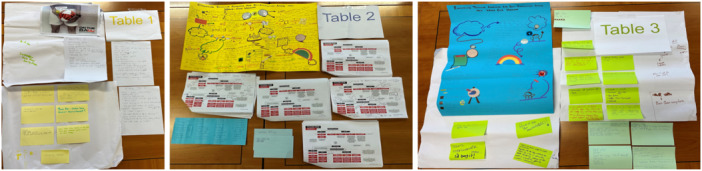
Written artefacts generated during the second World Café workshop.

The second WC workshop took place in July 2023 in an accessible venue. Participants provided written informed consent and completed a sociodemographic questionnaire. The host began the WC workshop with a short presentation explaining the purpose and process of the workshop. Participants were then given time to read and reflect on the ‘On the Ball’ campaign brief. After which, they were randomly assigned to three tables. They rotated tables at random for three 20‐min rounds of conversations. Each table had a scribe who took notes and a facilitator who focussed on one element of the campaign brief. The topic guide used by WC workshop facilitators on each of the three tables can be found in Supporting Information S1: File 1. Each table had audio recorders, drawing sheets, mind maps and artefact cards. Artefacts generated during the second WC workshop are presented in Figure 2. The workshop ended with a harvesting activity whereby facilitators and scribes provided a summary of the discussions that took place on their respective tables. Participants were invited to contribute to this activity. The workshop lasted around 2.5 h.

### 2.3 Data Analysis

Since this study uses a naturalistic inquiry, no pre‐existing frameworks were used to analyse the data [26]. Data were analysed using deductive thematic analysis [27]. Written participant responses and the scribes' notes were typed, and audio recordings were transcribed verbatim. Line‐by‐line coding was then conducted. Similar codes were collapsed and grouped to form subthemes. Themes linking the different subthemes were then created, guided by the three topic areas discussed during the WC workshop, hence the deductive nature of the analysis. Data were analysed by one researcher (V.N.S.) and crosschecked for accuracy and finalised in agreement with five senior academics and researchers (M.M.S., M.M.C., A.F., M.P.D. and J.H.).

## 3 Results

### 3.1 Participant Characteristics

In total, 20 participants contributed to the first and second WC workshops. Of those, 17 participated in the first WC workshop [22] and 13 in the second WC workshop. Characteristics of participants in the second WC workshop are presented in Table 1.

**Table 1:** Characteristics of participants in the second World Café workshop (*N*  =  13).

Characteristic	*n*	%
Age range (years)		
18–29	4	30.77
30–39	6	46.15
40–49	2	15.38
50–59	1	7.69
Gender		
Male	9	69.23
Female	3	23.08
Agender	1	7.69
Nationality		
Irish	11	84.62
Croatian	1	7.69
Spanish	1	7.69
Sexual orientation		
Gay	9	69.23
Heterosexual	3	23.08
Pansexual	1	7.69
Employment^a^		
Employed	12	92.31
Student	2	15.38
Unemployed	1	7.69
Current role^a^		
Member of an LGBTQ+ friendly organisation	8	61.54
Member of a sports club	5	38.46
Member of a men's health organisation	2	15.38
Member of a student body	2	15.38
Member of a governmental organisation	1	7.69
Testicular cancer survivor	1	7.69

^a^ Participants could choose more than one option.

### 3.2 Findings From the World Café Workshop

Findings are presented thematically in Table 2. While comments were predominantly positive, participants made recommendations to improve the campaign visuals:

*“*The red LL [in the logo] needs to be clear. That those are Ls. Some people thought it might look like a U.*”*

**Table 2:** Themes, subthemes and sample codes generated from the second World Café workshop, which aimed to refine and plan the launch of the ‘On the Ball’ campaign.

Theme	Subtheme	Sample codes
Campaign identity	Logo and design	Logo looks like BAUU as opposed to BALL. Letter L looks like J. Needs to be less sporty/masculine.
	Slogan	Overuse of the catchphrases in the image of the model. Rephrase ‘Have You Checked Your Testicles’.
	Instagram posts	Instagram page needs to be more colourful to grab attention. Instagram page should have stories of survivors.
Campaign delivery	Offline campaign delivery	Radio and television advertisements. One‐page summary document. Portable document format (PDF) document with many pages is not a good idea. Wary of downloading booklets. Interactive questionnaires. Ticking the box if self‐exam was conducted. Monitors/screens in universities.
	Online campaign delivery	TikTok Instagram X (formerly Twitter) Advertisements on dating apps. Questions using Instagram stories.
Campaign impact	Engaging the wider community/public	Include different influencers for younger and older and for the local community. Podcasters from Cork [city where the study was conducted]. Linking with community organisations/existing groups. Reach people working in warehouses (places with a large number of men). Customising campaign for people who drive or people who use trains. Other possible locations to share the campaign: City centre, universities, shopping centres, city library, public buildings, banks, hospitals, health centres, cafés and nearby clubs where there is larger population reach.
	Scaling up the campaign	Targeting different hubs—geographically distributing the campaign. Promotions during song contexts. Partnership with sports games/clubs. Linking in with festivals.
	Measuring campaign impact	Health Service Executive (HSE) report. Number of visits to general practitioners. Statistics and metrics. Percentage of early diagnosis. Number of shares on social media.

For some, the campaign poster featuring the torso of a man holding a tennis ball and a football symbolising one normal and one enlarged testis was too ‘sporty’ and ‘hyper‐masculinated’. As a result, they recommended more inclusivity and variety in the campaign imagery:

*“*You have another picture of a chef with two different ball things … like two different tomatoes. If that's gonna be a theme, I think that's so clever. Less sporty and less pink and red.*”*

As for the original campaign slogan ‘Have you Checked your Balls?’, participants recommended changing it to ‘Have you Checked Yours?’:

*“*‘Have you Checked Yours?’ might work better in the context of On the Ball. ‘Have you Checked Yours’ as opposed to ‘Your Balls.’ ‘Have you Checked Your Balls?’ is a bit repetitive.*”*

The sample social media posts enclosed within the brief were favoured by many participants. A few, however, believed that some posts were ‘lacking in personality’ and were ‘lifeless’:

*“*A change of colour and a different border around something [posts] would make it [campaign] way more inviting.*”*

As for the plan for campaign delivery, participants recommended delivering the campaign both, online and offline. Suggested online platforms included social media such as Instagram, TikTok and X (formerly Twitter), with preference for TikTok:

*“*TikTok's^TM^ probably your best bet, as opposed to Instagram^TM^. Twitter^TM^ nowadays is kind of going downhill in terms of the restrictions on it.*”*

The use of dating mobile phone applications like ‘Tinder’ and ‘Grindr’ was also recommended as part of online campaign delivery.

In terms of offline campaign delivery, the use of both, broadcast and print media, was recommended, with a preference for the former. Participants suggested advertising the campaign on the radio, television and monitors/screens in universities. As for print media, participants debated whether booklets are of value. Instead, they suggested a one‐page summary document.

Participants reflected on ways to achieve and measure campaign impact. They suggested reaching out to the wider community/public and targeting areas with a large number of men such as workplaces (e.g., warehouses), festivals, song contests, city centre, shopping centres, city libraries, public buildings, banks, hospitals, health centres, cafés and clubs:

*“*… I work with the lads in the warehouse. There's loads of them. You can sell them anything. And not because they're simple. They're not. But because they're so happy and appreciative that somebody took the time and said ‘if you give me a moment of your time. I want to talk to you about something and here's a sandwich.’ They're really happy about that. I mentioned [name of warehouse] purely because I know the company. They have 20 stores. They have warehouses across Ireland. Getting to those warehouses, maybe that's 400 men.*”*

Linking in with existing groups and community organisations was also recommended:

*“*… working with even the existing groups that are represented here today. Working with them to actually spread that kind of piece even within their own cohorts … some have stronger social media … some already have kind of existing platforms that can be used.*”*

In terms of measuring campaign impact, participants recommended capturing social media analytics such as the number of times the campaign was shared. They also recommended tracking the number of visits to general practitioners and national statistics on the incidence of testicular diseases:

*“*On a national level, the HSE [Health Service Executive, Ireland's main provider of public health and social care services] report. Just generally the waves of people inquiring with their GP [General Practitioner]. They could just automatically use that … you translate what the inquiries about particular cancers have gone up … you'd have GP figures for people who are reaching out with suspicions, or I found this, or I found that and then overall you should see early prevention numbers get better the more times you write those in.*”*

**Figure 3:**
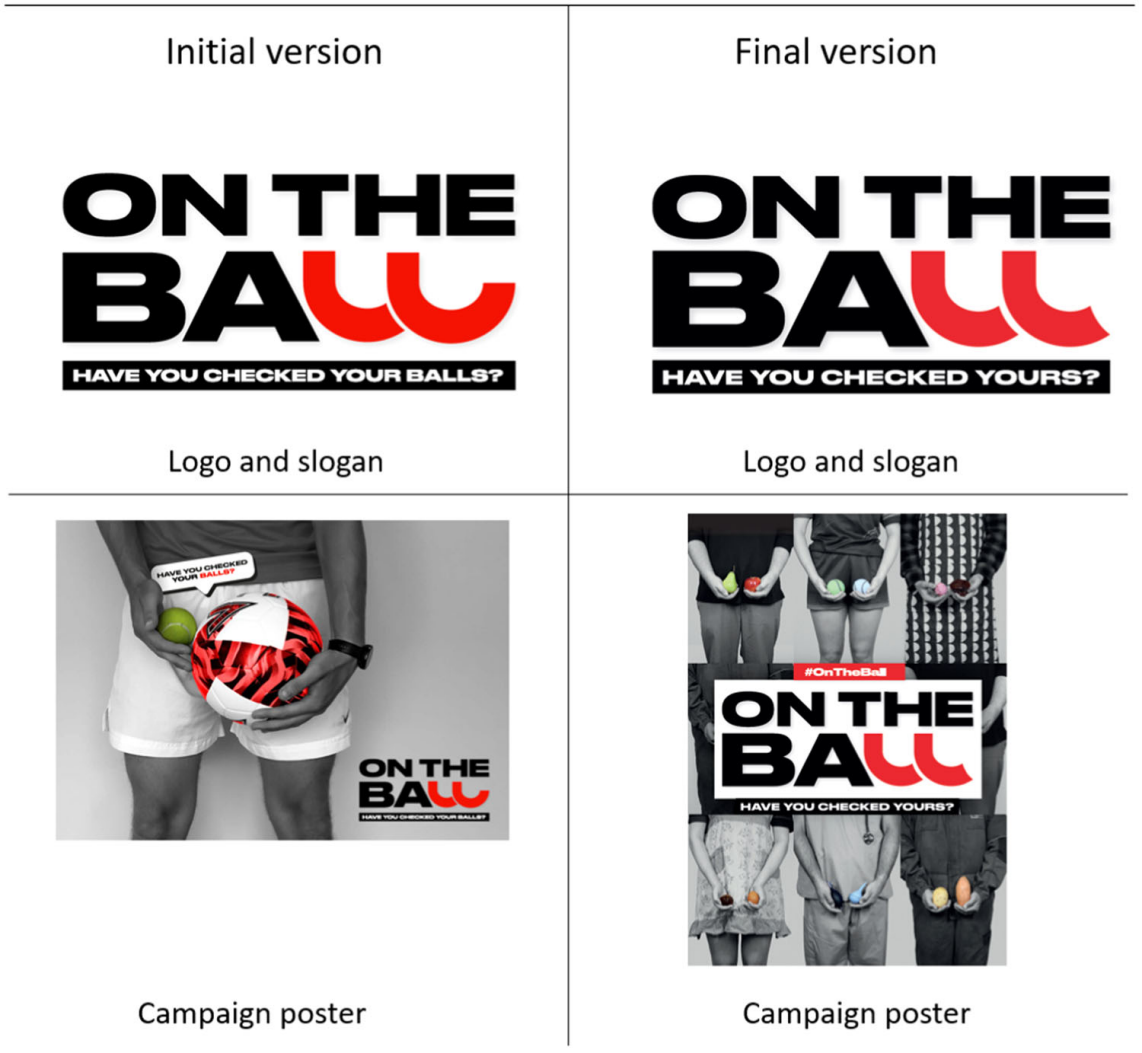
The initial version and the final version of the ‘On the Ball’ campaign.

The above recommendations were shared with the creative communications agency, and the campaign visuals were refined and finalised accordingly. For instance, the logo was edited, the slogan was shortened and imagery in the poster was made more inclusive. The initial and final versions of the campaign are presented in Figure 3.

### 3.3 Soft Launch of the ‘On the Ball’ Campaign

A soft launch of the ‘On the Ball’ campaign took place over two and a half days during the University's Sexual Health Awareness and Guidance (SHAG) week in October 2023. One exhibition stand was used on each day in areas with high student traffic. Each stand had large posters, pull‐up banners carrying the campaign identity and key campaign messages underpinned by the concept ‘testicular awareness’ and three ‘Ball Boxes’.

**Figure 4:**
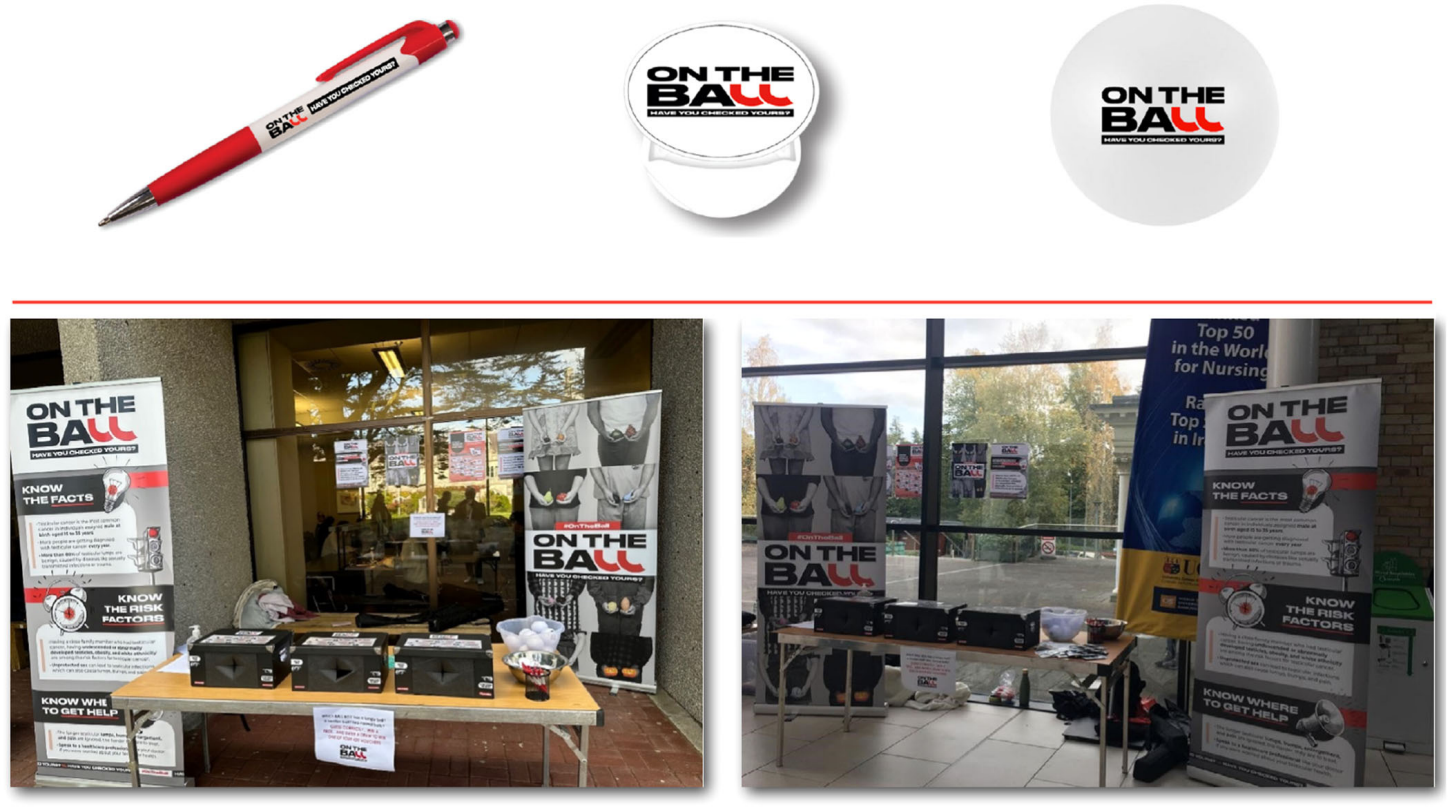
Photos from the soft launch of the ‘On the Ball’ campaign including campaign merchandise and the Ball Box novelty game.

Two research personnel attended the stand, providing information about testicular diseases and Ball Box game rules and tracking the number of students who visited the stands. Ball Boxes are novelty games designed based on recommendations from the first WC workshop [22]. Each box contained a pair of 3D printed testes enclosed within a soft elastic fabric, representing the scrotum. One box had a pair of normal testes, another box had one normal testis and one lumpy testis and the third box had one normal and one swollen testis. Students who approached the stands were asked to insert their hands inside each box and try to guess whether the testes were normal, swollen or lumpy. They were then asked to choose one of three prizes/merchandise namely a stress ball, a pen or a PopSocket (i.e., plastic circle attachable to the back of a mobile phone for a more comfortable/tighter grip). All merchandise carried the campaign logo ‘On the Ball’ and the slogan ‘Have You Checked Yours?’ There was also a draw for four gift vouchers. A total of 411 students approached the two stands and engaged with the various elements of the campaign. The two campaign stands, merchandise and Ball Boxes are presented in Figure 4.

## 4 Discussion

The ‘On the Ball’ campaign was codesigned and refined following two WC workshops with various community groups. Participants made recommendations to improve the campaign's visual identity, which is key to conveying campaign messages and fostering engagement, particularly since visual content is processed more rapidly than textual information [28]. A campaign's visual identity includes colour schemes, typography, imagery and design aesthetics, which play an important role in creating a long‐lasting memory of the campaign [29]. In the context of cancer awareness, a visually appealing campaign is more likely to encourage positive behaviour change and promote awareness [30].

Participants recommended delivering the ‘On the Ball’ campaign online using various social media platforms and dating mobile phone applications. At present, there are over 5.07 billion social media users, with a projected increase to more than 6 billion users in 2027 [31]. Social media remains one of the fastest means to communicate health information [32]. The use of social media platforms (e.g., Facebook, X, Instagram and TikTok) in health promotion campaigns in general and cancer awareness campaigns in particular has become increasingly prevalent due to the wide reach of such platforms. Using visual and interactive content in real time, as well as targeted messaging, such campaigns have the potential to promote cancer awareness and early detection. Indeed, evidence from two literature reviews suggests that social media and mobile health interventions have the potential to be effective in delivering interventions for cancer screening, prevention and management [33, 34].

However, in the age of misinformation and disinformation, the quality of social media posts on cancer awareness varies widely, ranging from evidence‐based content to misleading or inaccurate information. Indeed, studies evaluating the accuracy of information on genitourinary malignancies on various social media platforms found that the quality of the content was poor, and the prevalence of inaccurate or misleading information was high [35, 36, 37]. It was also found that misleading information was more likely to be shared than evidence‐based information [35].

Participants also suggested delivering the campaign offline using multiple platforms such as the radio, television, monitors/screens in universities and to a lesser extent print media. Offline communication is often regarded as a traditional way to deliver health information. It is suitable to reach out to individuals who do not use social media including older men and individuals with low levels of literacy/health literacy [38]. In fact, most interventions aimed at promoting testicular cancer awareness have effectively used offline communication such as PowerPoint presentations and videos [17].

Participants recommended several strategies to scale up the ‘On the Ball’ campaign and engage the wider community/public such as collaborations with influencers and community organisations. Collaborations with community organisations have the potential to enhance the communication of health messages and ensure the dissemination of accurate, evidence‐based information [39]. Moreover, engaging influencers or celebrities, particularly those with a lived experience of a testicular disease, can also strengthen campaign messages and enhance reach [30, 40]. Delivering the ‘On the Ball’ campaign in high‐traffic areas such as workplaces was also recommended to increase campaign reach. Workplace‐based interventions have the potential to reach a large segment of men, including those who are less likely to actively seek health information or access preventive care [41].

As for measuring the impact of the campaign, participants proposed tracking the number of visits to general practitioners, statistics relating to testicular diseases and social media analytics. All these strategies are supported by the wider literature [42, 43, 44]. The campaign's educational impact can also be measured by assessing changes in knowledge, awareness, help‐seeking intentions and testicular self‐examination behaviours [17]. Conducting postcampaign surveys or interviews to explore participants' feedback regarding campaign content, mode(s) of delivery and perceived effectiveness can inform future campaign refinement, implementation and evaluation [45].

## 5 Limitations

Thirteen individuals participated in the WC workshop, limiting the wider transferability of findings. Having four to five participants per table and using voice recorders could have affected participants' willingness to discuss their ideas openly. This risk was mitigated by creating a welcoming café‐like environment. As for the campaign launch, due to resource/budget limitations, we were unable to address all participants' recommendations, particularly delivering the campaign online, scaling it up and measuring its wider impact. Instead, a soft launch of the campaign was conducted in a university.

## 6 Conclusion

In conclusion, the ‘On the Ball’ campaign should be delivered both online and offline. This would help build the campaign profile, spread the campaign messages and reach out to a wider cohort. The process of online and offline campaign delivery ought to be investigated including campaign acceptability, adoption, appropriateness, cost, feasibility, fidelity, penetration and sustainability [46]. In line with participant recommendations and Medical Research Council guidance for complex intervention development and evaluation [47], future research is needed to explore online and offline campaign awareness, reach, engagement, effect on testicular awareness and effect on clinical outcomes including the incidence of testicular diseases.

## Author Contributions

**Mohamad M. Saab**: conceptualisation, investigation, funding acquisition, writing–original draft, methodology, formal analysis, project administration, data curation, resources. **Varsha N. Shetty**: investigation, writing–original draft, formal analysis, project administration, data curation. **Megan McCarthy**: investigation, writing–original draft, formal analysis, data curation. **Martin P. Davoren**: conceptualisation, investigation, methodology, writing–review and editing. **Angela Flynn**: conceptualisation, investigation, methodology, writing–review and editing. **Ann Kirby**: conceptualization, writing–review and editing. **Steve Robertson**: conceptualisation, methodology, writing–review and editing. **Gillian W. Shorter**: conceptualisation, methodology, writing–review and editing. **David Murphy**: conceptualisation, methodology, writing–review and editing. **Michael J. Rovito**: conceptualisation, methodology, writing–review and editing. **Frances Shiely**: conceptualisation, methodology, writing–review and editing. **Josephine Hegarty**: conceptualisation, investigation, writing–review and editing, methodology.

## Ethics Statement

This study received ethical approval from the Social Research Ethics Committee at University College Cork (Log 2022‐227).

## Consent

All participants were required to provide written informed consent before data collection.

## Conflicts of Interest

The authors declare no conflicts of interest.

## Supporting information

Supporting information.

## Data Availability

The data that support the findings of this study are available from the corresponding author upon reasonable request.

## References

[1] A. Znaor , N. E. Skakkebaek , E. Rajpert‐De Meyts , et al., “Global Patterns in Testicular Cancer Incidence and Mortality in 2020,” International Journal of Cancer 151, no. 5 (2022): 692–698, 10.1002/ijc.33999. 35277970 PMC12980092

[2] S. M. Stevenson and W. T. Lowrance , “Epidemiology and Diagnosis of Testis Cancer,” Urologic Clinics of North America 42, no. 3 (2015): 269–275, 10.1016/j.ucl.2015.04.001. 26216814

[3] M. P. Laguna , P. Albers , F. Algaba , et al., “Testicular Cancer.” In *EAU Annual Congress Amsterdam* (2020). https://www.researchgate.net/profile/Ricardo-Leao-2/publication/340681546_EAU_GUIDELINES_ON_TESTICULAR_CANCER_Epidemiology_eatiology_and_pathology_Histological_classification/links/5eb07106a6fdcc7050a8d669/EAU-GUIDELINES-ON-TESTICULAR-CANCER-Epidemiology-eatiology-and-pathology-Histological-classification.pdf.

[4] N. H. Hanna and L. H. Einhorn , “Testicular Cancer—Discoveries and Updates,” New England Journal of Medicine 371, no. 21 (November 2014): 2005–2016. 25409373 10.1056/NEJMra1407550

[5] S. M. Stevenson and W. T. Lowrance , “Epidemiology and Diagnosis of Testis Cancer,” Urologic Clinics of North America 42, no. 3 (August 2015): 269–275. 26216814 10.1016/j.ucl.2015.04.001

[6] T. H. Trojian , T. S. Lishnak , and D. Heiman , “Epididymitis and Orchitis: An Overview,” American Family Physician 79, no. 7 (2009): 583–587. 19378875

[7] S. M. Wampler and M. Llanes , “Common Scrotal and Testicular Problems,” Primary Care: Clinics in Office Practice 37, no. 3 (2010): 613–626, 10.1016/j.pop.2010.04.009. 20705202

[8] R. I. Clavijo , R. Carrasquillo , and R. Ramasamy , “Varicoceles: Prevalence and Pathogenesis in Adult Men,” Fertility and Sterility 108, no. 3 (2017): 364–369, 10.1016/j.fertnstert.2017.06.036. 28865534

[9] E. Ringdahl and L. Teague , “Testicular Torsion,” American Family Physician 74, no. 10 (2006): 1739–1743. 17137004

[10] C. E. Bayne , J. Villanueva , T. D. Davis , H. G. Pohl , and H. G. Rushton , “Factors Associated With Delayed Presentation and Misdiagnosis of Testicular Torsion: A Case–Control Study,” The Journal of Pediatrics 186 (2017): 200–204, 10.1016/j.jpeds.2017.03.037. 28427778

[11] L. A. Mcguinness , S. Obeidat , B. Hickerton , and R. Long , “Has Increasing Public Health Awareness Influenced the Size of Testicular Tumours Among Adult Populations Over the Last 40 Years?” Journal of Public Health 39, no. 1 (March 2017): 90–94. 26944075 10.1093/pubmed/fdw014

[12] M. M. Saab , M. Landers , and J. Hegarty , “Promoting Testicular Cancer Awareness and Screening: A Systematic Review of Interventions,” Cancer Nursing 39, no. 6 (2016): 473–487, 10.1097/NCC.0000000000000333. 26859280

[13] M. Saab , M. Landers , and J. Hegarty , “Testicular Cancer Awareness and Screening Practices: A Systematic Review,” Oncology Nursing Forum 43, no. 1 (2016): E8–E23, 10.1188/16.ONF.E8-E23. 26679456

[14] M. M. Saab , M. P. Davoren , A. Murphy , et al., “Promoting Men's Awareness, Self‐Examination, and Help‐Seeking for Testicular Disorders: A Systematic Review of Interventions,” HRB Open Research 1 (2018): 16, 10.12688/hrbopenres.12837.2. 32002508 PMC6973532

[15] M. M. Saab , M. Landers , and J. Hegarty , “Males' Awareness of Benign Testicular Disorders: An Integrative Review,” American Journal of Men's Health 12, no. 3 (2018): 556–566, 10.1177/1557988315626508. PMC5987954 26783155

[16] M. J. Rovito , C. Cavayero , J. E. Leone , and S. Harlin , “Interventions Promoting Testicular Self‐Examination (TSE) Performance: A Systematic Review,” American Journal of Men's Health 9, no. 6 (2015): 506–518, 10.1177/1557988314555360. 25359870

[17] M. M. Saab , M. P. Davoren , A. Murphy , et al., “Promoting Men's Awareness, Self‐examination, and Help‐Seeking for Testicular Disorders: A Systematic Review of Interventions,” HRB Open Research 1 (2023): 16, 10.12688/hrbopenres.12837.3. 32002508 PMC6973532

[18] M. M. Saab , M. Landers , E. Cooke , D. Murphy , M. Davoren , and J. Hegarty , “Enhancing Men's Awareness of Testicular Disorders Using a Virtual Reality Intervention: A Pre–Post Pilot Study,” Nursing Research 67, no. 5 (September 2018): 349–358. 30059354 10.1097/NNR.0000000000000303

[19] S. A. Kirjava , D. Rawal , A. Xia , and M. Moshin , “Contemporary LGBTQ+ Content That Should be Included in Allied Health Professions Education,” Discover Education 2, no. 1 (2023): 6, 10.1007/s44217-023-00029-y.

[20] J. S. Hocking , W. M. Geisler , and F. Y. S. Kong , “Update on the Epidemiology, Screening, and Management of Chlamydia trachomatis Infection,” Infectious Disease Clinics of North America 37, no. 2 (2023): 267–288, 10.1016/j.idc.2023.02.007. 37005162

[21] M. M. Saab , J. Hegarty , and M. Landers , “Testicular Awareness: The What, the Why, and the How,” International Journal of Men's Social and Community Health 2, no. 1 (2019): e1–e10, 10.22374/ijmsch.v2i1.16.

[22] M. M. Saab , V. N. Shetty , M. McCarthy , et al., “Promoting ‘Testicular Awareness’: Co‐design of an Inclusive Campaign Using the World Café Methodology,” Health Expectations 27, no. 1 (2024): e13898. 10.1111/hex.13898 PMC10726274 37877701

[23] B. Smith , O. Williams , L. Bone , and M. S. W. C. Collective , “Co‐Production: A Resource to Guide Co‐producing Research in the Sport, Exercise, and Health Sciences,” Qualitative Research in Sport, Exercise and Health 15, no. 2 (2023): 159–187, 10.1080/2159676X.2022.2052946.

[24] J. Monforte , J. Netherway , and B. Smith , “The World Café Is an Unmethod Within Co‐Produced Research,” Qualitative Research in Psychology 20, no. 3 (2023): 398–419, 10.1080/14780887.2023.2239728.

[25] “The World Cafe,” World Cafe Method (2015), https://theworldcafe.com/key-concepts-resources/world-cafe-method/.

[26] M. Sandelowski , “Whatever Happened to Qualitative Description?” Research in Nursing & Health 23, no. 4 (August 2000): 334–340. 10940958 10.1002/1098-240x(200008)23:4<334::aid-nur9>3.0.co;2-g

[27] V. Braun and V. Clarke , “Thematic Analysis,” in APA Handbook of Research Methods in Psychology, Vol 2: Research Designs: Quantitative, Qualitative, Neuropsychological, and Biological, eds. H. Cooper , P. M. Camic , D. L. Long , A. T. Panter , D. Rindskopf , and K. J. Sher (Washington, DC: American Psychological Association, 2012), 57–71, 10.1037/13620-004.

[28] P. S. Houts , C. C. Doak , L. G. Doak , and M. J. Loscalzo , “The Role of Pictures in Improving Health Communication: A Review of Research on Attention, Comprehension, Recall, and Adherence,” Patient Education and Counseling 61, no. 2 (May 2006): 173–190. 16122896 10.1016/j.pec.2005.05.004

[29] K. Metral , “The Power of Color Psychology in Web Design” (2024), https://cosmicostudios.medium.com/the-power-of-color-psychology-in-web-design-b798e956797a#:~:text=Colors%20have%20the%20power%20to,user%20engagement%20and%20conversion%20rates.

[30] M. M. Saab , C. Kilty , B. Noonan , et al., “Public Health Messaging and Strategies to Promote ‘SWIFT’ Lung Cancer Detection: A Qualitative Study Among High‐Risk Individuals,” Journal of Cancer Education 37 (2022): 1026–1035. 33131021 10.1007/s13187-020-01916-w PMC9399198

[31] “Social Media—Statistics & Facts,” Statista (2024), https://www.statista.com/topics/1164/social-networks/#editorsPicks.

[32] J. Chen and Y. Wang , “Social Media Use for Health Purposes: Systematic Review,” Journal of Medical Internet Research 23, no. 5 (2021): e17917. 33978589 10.2196/17917 PMC8156131

[33] C. J. Han , Y. J. Lee , and G. Demiris , “Interventions Using Social Media for Cancer Prevention and Management: A Systematic Review,” Cancer Nursing 41, no. 6 (November 2018): E19–E31. 10.1097/NCC.0000000000000534 PMC5787052 28753192

[34] A. Ruco , F. Dossa , J. Tinmouth , et al., “Social Media and mHealth Technology for Cancer Screening: Systematic Review and Meta‐analysis,” Journal of Medical Internet Research 23, no. 7 (July 2021): e26759. 34328423 10.2196/26759 PMC8367160

[35] M. Alsyouf , P. Stokes , D. Hur , A. Amasyali , H. Ruckle , and B. Hu , “‘Fake News' in Urology: Evaluating the Accuracy of Articles Shared on Social Mediain Genitourinary Malignancies,” BJU International 124, no. 4 (2019): 701–706, 10.1111/bju.14787. 31044493

[36] M. B. Duran and Y. Kizilkan , “Quality Analysis of Testicular Cancer Videos on YouTube,” Andrologia 53, no. 8 (2021): e14118, 10.1111/and.14118. 34009641

[37] F. Di Bello , C. Collà Ruvolo , S. Cilio , et al., “Testicular Cancer and Youtube: What Do You Expect From a Social Media Platform?” International Journal of Urology 29, no. 7 (2022): 685–691, 10.1111/iju.14871. 35318754

[38] F. J. Drummond , M. Reidy , C. von Wagner , et al., “Health Literacy Influences Men's Active and Passive Cancer Information Seeking,” Health Literacy Research and Practice 3, no. 3 (2019): 147. 10.3928/24748307-20190430-01 PMC6685514 31410385

[39] J. Macnamara and M. Camit , “Effective CALD Community Health Communication Through Research and Collaboration: An Exemplar Case Study,” Communication Research and Practice 3, no. 1 (January 2017): 92–112.

[40] M. S. Patel , J. A. Halpern , A. S. Desai , M. K. Keeter , N. E. Bennett , and R. E. Brannigan , “Success of Prostate and Testicular Cancer Awareness Campaigns Compared to Breast Cancer Awareness Month According to Internet Search Volumes: A Google Trends Analysis,” Urology 139 (May 2020): 64–70. 32001306 10.1016/j.urology.2019.11.062

[41] A. Dolan , V. Staples , S. Summer , and G. L. Hundt , “‘You Ain't Going to Say I've Got a Problem Down There’: Workplace‐Based Prostate Health Promotion With Men,” Health Education Research 20, no. 6 (December 2005): 730–738. 15878937 10.1093/her/cyh033

[42] D. Feldman‐Stewart , C. Tong , and M. D. Brundage , “Evaluation of a Widely Available Patient Decision Aid for the Treatment of Prostate Cancer,” Patient Education and Counseling 101, no. 10 (October 2018): 1761–1766. 29729858 10.1016/j.pec.2018.04.015

[43] J. P. Struck , F. Siegel , M. W. Kramer , et al., “Substantial utilization of Facebook, Twitter, YouTube, and Instagram in the Prostate Cancer Community,” World Journal of Urology 36 (2018): 1241–1246. 29523948 10.1007/s00345-018-2254-2

[44] M. M. Saab , S. FitzGerald , B. Noonan , et al., “Promoting Lung Cancer Awareness, Help‐Seeking and Early Detection: A Systematic Review of Interventions,” Health Promotion International 36, no. 6 (2021): 1656–1671. 33647930 10.1093/heapro/daab016 PMC8699397

[45] M. M. Saab , M. Landers , E. Cooke , D. Murphy , and J. Hegarty , “Feasibility and Usability of a Virtual Reality Intervention to Enhance Men's Awareness of Testicular Disorders (E‐MAT),” Virtual Reality 23 (June 2019): 169–178.

[46] R. E. Glasgow , T. M. Vogt , and S. M. Boles , “Evaluating the Public Health Impact of Health Promotion Interventions: The RE‐AIM Framework,” American Journal of Public Health 89, no. 9 (1999): 1322–1327, 10.2105/AJPH.89.9.1322. 10474547 PMC1508772

[47] K. Skivington , L. Matthews , S. A. Simpson , et al., “A New Framework for Developing and Evaluating Complex Interventions: Update of Medical Research Council Guidance,” BMJ 30 (2021): n2061, 10.1136/bmj.n2061. PMC8482308 34593508

