# Supplementary material for: Codesign and Launch of ‘On the Ball’: An Inclusive Community‐Based ‘Testicular Awareness’ Campaign
Source: Health Expect. 2024 Jun 14;27(3):e14100. doi: 10.1111/hex.14100 (PMC11176580; doi:10.1111/hex.14100)
Supplement: Supplementary file 1 — Supporting information. [file HEX-27-e14100-s001.docx]

**Supporting Information File 1.** Topic guide used by facilitators on each of the three World Café workshop tables.

| **TABLE 1.** |
| --- |
| **Questions** |
| Q1. Take some time to look at the campaign identity, design, logo, and catchphrase. What do you think about the message? Is it clear? |
| Q2. What do you like the most about the campaign? |
| Q3. What do you like the least about the campaign? |
| Q4. What would you change about the campaign? |
| **TABLE 2.** |
| **Questions** |
| Q1. In front you is the campaign brief. Phase 1 or “reach” will create a strong foundation for the rest of the campaign and will be delivered digitally using mainly social media. Take some time to go over Phase 1. What do you think? What would you change? |
| Q2. Phase 2 or “interact” will bring the campaign offline such as on campus to stimulate in-person conversations. Take some time to go over Phase 2. What do you think? What would you change? |
| Q3. In front of you are three black boxes containing a set of testicles, some are normal, and others are either swollen or lumpy. Take some time to check those. What do you think about the look and feel of the box and its content? What would you change? Where do you think this box can be used? |
| **TABLE 3.** |
| **Questions** |
| Q1. Campaign Phase 3 or “engage” involves the dissemination of the campaign among a larger audience and getting various collaborators and influencers on board to help grow and spread the campaign message wider. How do you think this can be achieved? |
| Q2. Due to our limited budget, we are limiting the campaign to a certain geographical area (i.e., Cork). However, we are thinking about developing and sustaining the campaign in the long-term. How can we scale up the campaign? How can we sustain the digital presence of the campaign? |
| Q3. If we are to seek further funding to develop and deliver the campaign at a wider scale, where shall we seek funding from? Who do we approach? |
| Q4. How can we measure the impact of the campaign? In other words, how do we know whether the campaign works? |
